# Human mobility trends during the early stage of the COVID-19 pandemic in the United States

**DOI:** 10.1371/journal.pone.0241468

**Published:** 2020-11-09

**Authors:** Minha Lee, Jun Zhao, Qianqian Sun, Yixuan Pan, Weiyi Zhou, Chenfeng Xiong, Lei Zhang

**Affiliations:** Department of Civil and Environmental Engineering, Maryland Transportation Institute, University of Maryland, College Park, Maryland, United States of America; Faculty of Science, Ain Shams University (ASU), EGYPT

## Abstract

In March of this year, COVID-19 was declared a pandemic, and it continues to threaten public health. This global health crisis imposes limitations on daily movements, which have deteriorated every sector in our society. Understanding public reactions to the virus and the non-pharmaceutical interventions should be of great help to fight COVID-19 in a strategic way. We aim to provide tangible evidence of the human mobility trends by comparing the day-by-day variations across the U.S. from January 2020 to early April 2020. Large-scale public mobility at an aggregated level is observed by leveraging mobile device location data and the measures related to social distancing. Our study captures spatial and temporal heterogeneity as well as the sociodemographic variations and teleworking trends regarding the pandemic propagation and the non-pharmaceutical mobility interventions. All metrics adapted capture decreased public movements after the national emergency declaration. The population staying home has increased in all states before the stay-at-home mandates implemented and becomes more stable after the order with a smaller range of fluctuation. The public had been taking active responses, voluntarily staying home more, to the in-state confirmed cases while the stay-at-home orders stabilize the variations. As the estimated teleworking rates also continue to incline throughout the study period, the teleworking trend can be another driving factor for the growing stay-at-home population. We confirm that there exists overall mobility heterogeneity between the income or population density groups. The study suggests that public mobility trends are in line with the government message urging to stay home. We anticipate our data-driven analysis offers integrated perspectives and serves as evidence to raise public awareness and, consequently, reinforce the importance of social distancing while assisting policymakers.

## Introduction

Historically, the year 2020 will be remembered for the global battle against an invisible enemy. Since the emergence of the novel coronavirus (COVID-19) in December 2019 in Wuhan, China, the world is experiencing unprecedented phenomena [1]. In March of this year, COVID-19 was declared a pandemic by the World Health Organization (WHO), and emergency measures have been internationally implemented as the outbreak continues to threaten public health. As of April 10, 2020, there were almost 1.7 million worldwide confirmed cases of COVID-19, with the United States accounting for over 500,000 cases, or around 30% of overall infections around the world [2]. As a result, over 40 American states have instituted stay-at-home orders, making quarantine and social distancing the new norm for the majority of the U.S. population.

Interdisciplinary research has been actively conducted to mitigate the spread of COVID-19 and its adverse impacts on society. Epidemiologic measurements have been explored to identify the dynamics of disease regarding the spread risk and the effect of human mobility [3–10]. Besides, since the mobility restrictions are considered as a critical factor to prevent the disease spread, studies have assessed its impact [11–16]. Non-pharmaceutical observations are also proven to be effective data sources to have an integrated perspective. Especially big data allow for increased understanding of human behavior changes in response to the spread of the virus. One recent study captures the dissemination of COVID-19 information in relation to the outbreak progression from medical-oriented social media sources [17]. Other studies employing data-driven methodologies have also been introduced to estimate the negative impacts on various sectors such as the economy, public health, and human mobility [18–23]. In particular, mobility data have been identified as being especially relevant, and researchers have provided in-depth knowledge on how to leverage mobile device location data for analyzing COVID-19 propagation [24–26]. Technology companies have presented insights on mobility trends by exploiting location data as well [27–29].

As revealed from literature, practicing social and physical distancing and timely decisions on mobility interventions are crucial to slow the pandemic. The key factor is those classical approaches, including social distancing, quarantine, and mobility interventions, as no treatment is currently available [13]. Since little is known about the gap between social distance advocacy and the actual practices among the general populace, however, comprehensive studies have been introduced that examine a broad spectrum of COVID-19 trends, including government action timelines and social distancing practices using mobility data [24, 30–34]. While the focus of these latest studies lies on the social, political, and economic aspects regarding the pandemic, there is still scant research that scrutinizes various mobility metrics and focuses mainly on spatial and temporal mobility variations and travel behavior. Realizing the urgent needs on understanding mobility trends amid the pandemic, we aim to quantify changes in human mobility to provide tangible and intuitive evidence on individual and governmental efforts to migrate the spread. Our study explores daily travel behavior across the U.S. based on multiple mobility metrics, including stay-home population, out-of-county trips, work trips, non-work trips, and person miles traveled. To the best of our knowledge, this is one of the pioneering big data-driven studies on COVID-19 that adapt unique and novel data integrated from multiple data providers to improve the data quality. The goal of the study is two-fold. First, we explore large-scale public mobility patterns and the existence of heterogeneity across the nation by leveraging mobile device location data. It covers temporal trend analysis before and after the emergence of COVID-19 and mobility interventions and geospatial trend analysis at both nation and state levels in the U.S. Second, sociodemographic group comparisons and statewide teleworking trends are examined. While this paper does not intend to establish complete guidance on how governments or similar bodies should respond, the hope is to share our observations and findings to provide an integrated perspective on the public mobility reactions before and during the pandemic.

## Methods

### Data sources

The University of Maryland COVID-19 Impact Analysis Platform aggregates mobile device location data from more than 100 million anonymized sample devices each month [34]. The aggregated location data are then integrated with COVID-19 case data from John Hopkins University and census population data to monitor the mobility trends in the United States. The metrics produced from the data are provided only in aggregated forms at the county, state, and national levels.

### Data analysis

**Fig 1:**
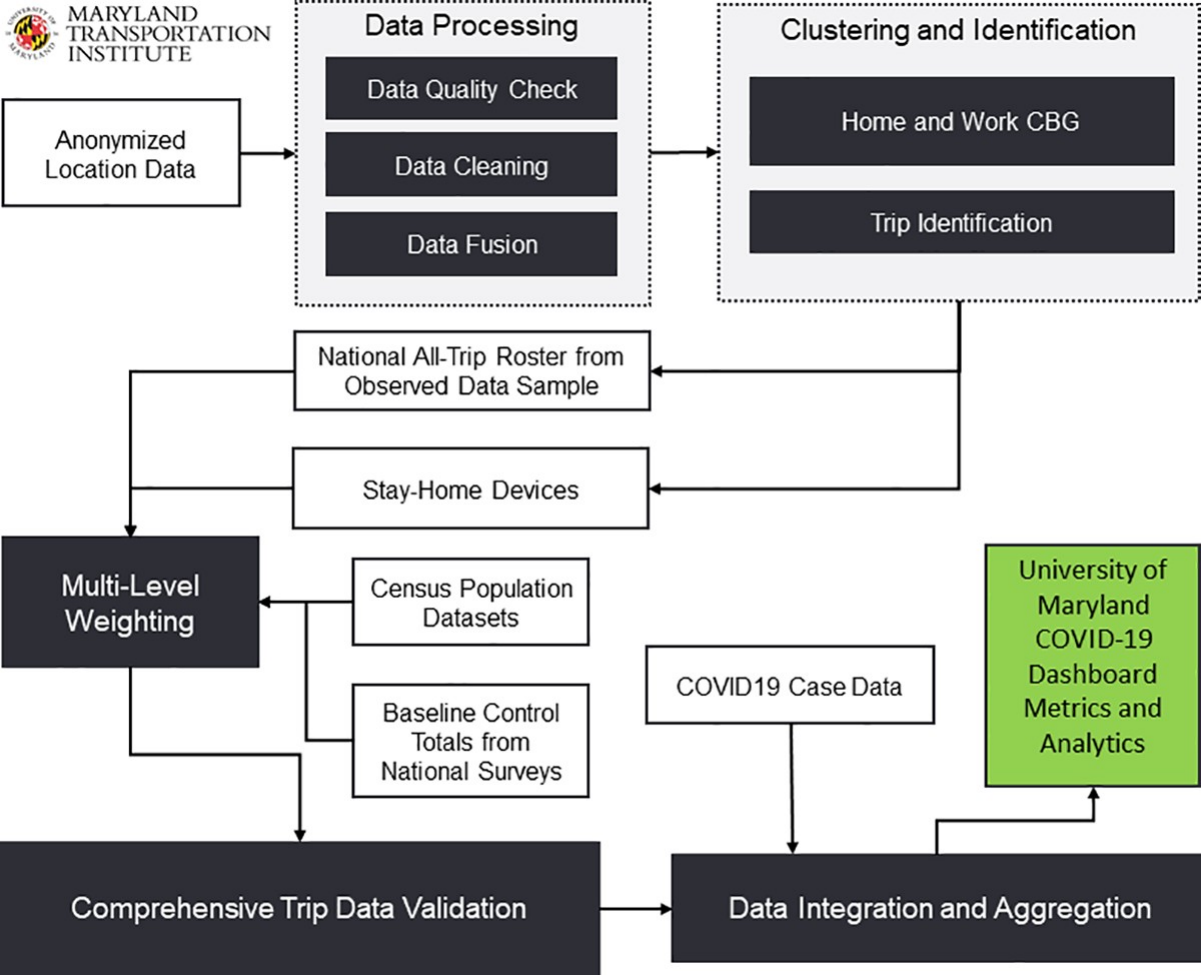
Data processing and methodology framework. Reprinted from [34, 36] under a CC BY license, with permission from Zhang L et al., original copyright 2020.

The research team first integrates and preprocesses person and vehicle movement data to improve the quality of our mobile device location data panel, followed by the trip identification process (Fig 1). Second, location points from all devices are clustered into activity locations from which potential candidates for the home and work locations are detected. Both temporal and spatial features for the entire activity locations are considered to identify home and work census block groups (CBGs). This step includes calculating cluster attributes such as total dwell time, maximum dwell time, average dwell time, and total visits frequency and device attributes such as the number of clusters and average cluster dwell time. Once the cluster locations are labeled with main point of interest, we identify the home and work CBGs and work types based on a set of calibrated heuristics. Third, additional trip information, including trip origin, destination, departure time, and arrival time, is identified based on previously developed and validated algorithms [35]. The condition of staying at home is defined if an anonymized individual in the sample does not travel farther than one mile from home. In the next step, we expand the sample data to the population level by incorporating a multi-level weighting procedure to have results that represent the entire population in a nation, state, or county. The data sources and algorithms implemented are validated based on various datasets such as the National Household Travel Survey (NHTS) and American Community Survey (ACS) and previously peer-reviewed by an external expert panel [35]. Lastly, mobility metrics are integrated with other data sources, such as COVID-19 cases and population. Table 1 summarizes the metrics we adapt in each analysis: national, statewide, sociodemographic groupwise, and teleworking trend. The whole set of metrics can be consulted in the platform and additional details of the methodology can be found in a separate paper by the authors [34, 36].

**Table 1:** A Summary of metrics adapted from the COVID-19 Impact Analysis Platform.

Metrics	Description	Nation	State	Socio-demographic	Teleworking
% staying home	Percentage of residents staying at home	√	√	√	
miles traveled per person	Average person-miles traveled on all modes	√	√	√	
% out-of-county trips	The percent of all trips taken that travel out of a county	√		√	
number of trips per person	Average number of trips taken per person	√			
number of work trips/person	Number of work trips per person where a work trip is defined as going to or coming from a fixed work location	√		√	√
number of non-work trips/person	Number of non-work trips per person (e.g. grocery stores, restaurants, etc.)	√			
number of COVID-19 cases	Number of confirmed COVID-19 cases from the Johns Hopkins University’s GitHub repository [37]	√	√	√	

### Spatiotemporal trend analysis

We explore the mobility variations regarding the COVID-19 progression and government stay-at-home orders by applying the metrics closely related to social distancing. Our trend analysis design can be categorized into four types: 1) nationwide; 2) statewide; 3) sociodemographic groupwise; and 4) teleworking trends. The mobility metrics applied are marked in Table 1, respectively. Temporal range covers from January 6, 2020 to April 9, 2020, while weekends are excluded to eliminate noises.

The nationwide trends are examined by applying a 3-day moving average method for all mobility metrics. The statewide trend analysis compares states with two types of timelines. The first is universal timelines: 1) benchmark week (February 3—February 16) and most recent week (April 6—April 12). All 50 states are considered in these timelines. The second type is stay-at-home order timelines: 1) one week before the order and 2) one week after the order which vary per state and are applied to 44 states with the order implemented as of April 2. Then the statewide analysis further examines the public reaction stability based on one measure, the percentage of people staying home, which we believe to have a high correlation with social distancing. The stability is evaluated by the mobility variations across time and states with a range and standard deviation, where higher values indicate lower stability. The range is to show raw spatial variations across states and temporal variations within states, including extreme values, and the standard deviation is to measure how disperse mobility points are.

The sociodemographic group comparison endeavors how these features influence the mobility patterns amid COVID-19. During the preliminary analysis, four features are considered: percentage of the middle-aged population (35 years old and over); percentage of older people (65 years old and over); median household income; and population density in persons per mile of land area. States are classified into two groups by each feature (higher: 25 states and lower: 26 states). In this paper, the result section delivers two comparison results from the median income and population density groups, which show a unique clustering nature. In contrast, the other two age-related features do not.

## Results and discussion

### Nationwide trends

**Fig 2:**
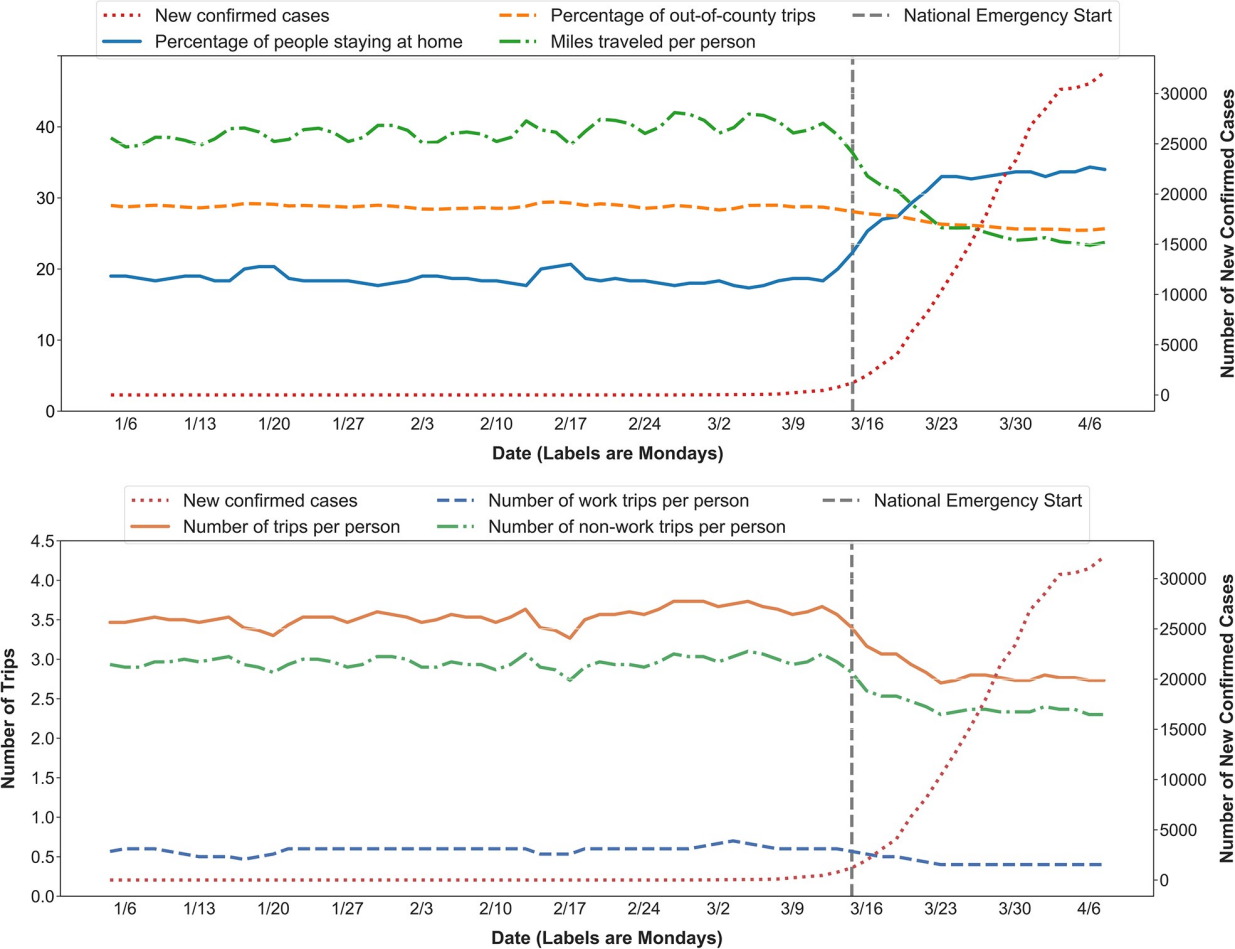
National trends on mobility measurements.

A large number of people have decreased their daily movements: the percentage of people staying home rapidly increases from 20% on regular days (benchmark week) to 35% after the outbreak (most recent week); out-of-county trips decreases from 28% to 23%; average trip distance drops from 40 miles to 23 miles; and the number of trips per person decreases from 3.7 (3.1 non-work trips and 0.6 work trips) to 2.7 (2.3 non-work trips and 0.4 work trips) trips (Fig 2). The out-of-county trips might seem to present trivial changes in Fig 2, compare to other metrics. This is because the percentage changes are illustrated to avoid excessive magnitude of absolute value, which reduced from 201 million to 151 million daily trips after March 13. The mobility trends change quickly around March 13 when the national emergency is declared, which is indicated by a grey bar in Fig 2, in accordance with the rapid increase of COVID-19 cases. This observation could have occurred since the emergency declaration raised public awareness on the pandemic, helped the broader spread of the information related to COVID-19, and encouraged more people to reduce mobility.

### Statewide trends

**Fig 3:**
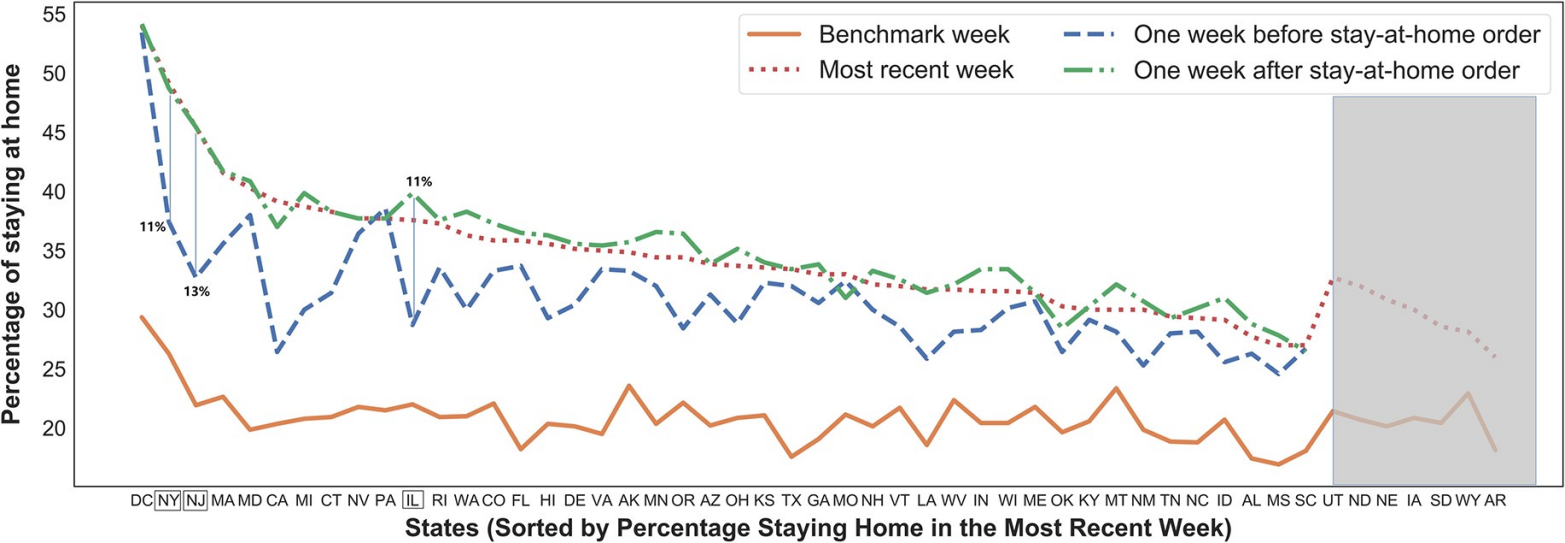
Statewide trends on the percentage of people staying home.

Fig 3 shows the percentage of people staying home in the highest order on the x-axis, while the grey shade deemphasizes states without the mandate. In the most recent week, the District of Columbia maintains the highest rate of people staying home (54%), followed by New York (49%) and New Jersey (45%). Three states with the lowest rates are Mississippi (27%), South Carolina (27%), and Arkansas (26%). In terms of changes between the week before and after the order, three states with the highest increase are New Jersey (+13%), New York (+11%), and Illinois (+11%) as marked with a green box in Fig 3. The lowest changes belong to Kentucky (+1.2%), Maine (+0.7%), and South Carolina (-0.2%). The average percentage increase is +4.3% between one week after and one week before the order.

**Fig 4:**
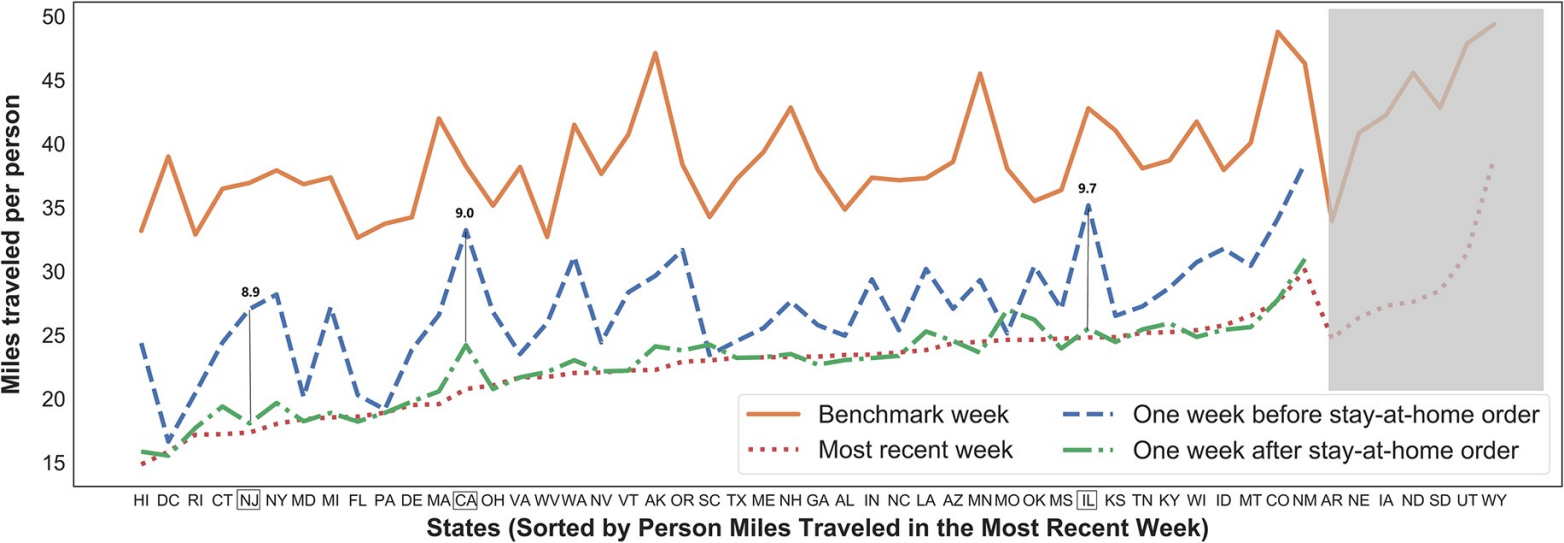
Statewide trends on miles traveled per person.

Stay-at-home orders also result in trip distance reduction (Fig 4). Hawaii records the lowest miles traveled per person (4.9 mi.), followed by The District of Columbia (15.8 mi.), and Rhode Island (17.2 mi.). Three states with the highest miles traveled are Wyoming (39.0 mi.), Utah (31.4 mi.), and New Mexico (30.1 mi.). The highest decline after the order is observed in Illinois (-9.7 mi.), California (-9.0 mi.), and New Jersey (-8.9 mi.), which are marked with a green box in Fig 4. In comparison, the lowest changes are found in Missouri (+1.9 mi.), South Carolina (+0.9 mi.), and Pennsylvania (-0.3 mi.). The stay-at-home order leads to four miles distance reduction on average.

**Fig 5:**
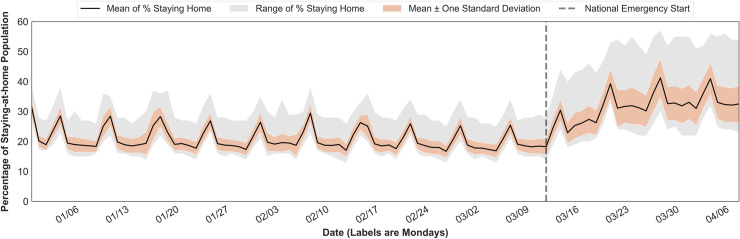
Temporal variations on the percentage of people staying home for all states.

**Fig 6:**
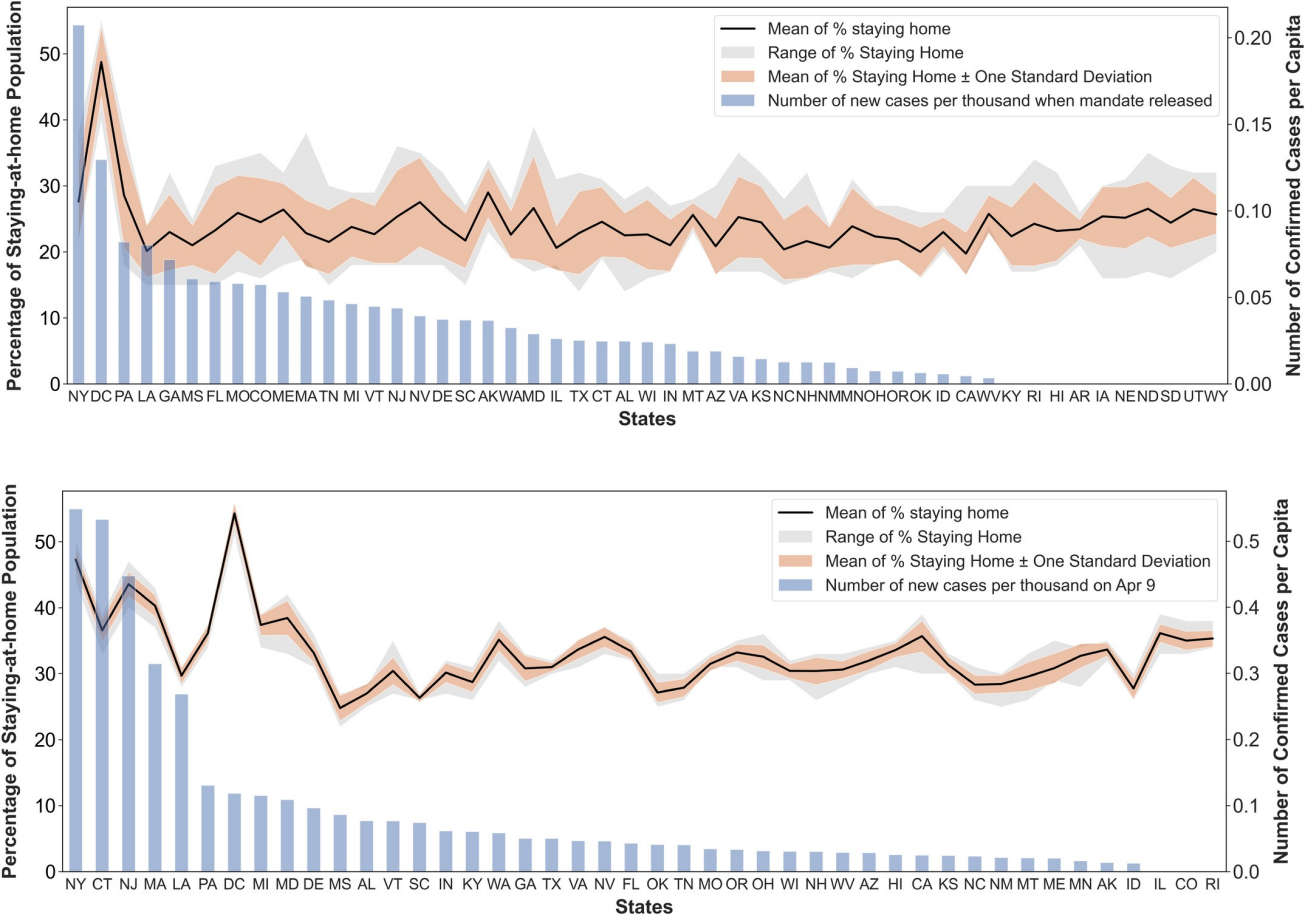
Statewide variations on the percentage of people staying at home by two timelines. (a) Percentage of people staying home between the first confirmed case and stay-at-home order (upper); (b) Percentage of people staying home after the stay-at-home order (lower).

To further evaluate the public reaction stability, we choose the percentage of people staying home statistics. Even though the national average of populations staying home has become higher, there emerges a larger temporal variance in the reactions of all the states after mid-March (Fig 5). It refers to a decrease in spatial stability indicating that social distancing practice varies across states possibly due to various virus spread status (Fig 5). The pre-pandemic range of the national staying-at-home population was from 17% to 31% with an average of 20%. Then it has inclined to the range of 23% and 43% with an average of 33% after March 13, 2020. We also compared the statewide public reaction stability based on two different pandemic stages. The first stage is between the temporal range from the date of the first COVID-19 case confirmed to the mandatory stay-at-home order issued in each state (Fig 6A). The second stage is after the order was released (Fig 6B). For states without the mandates, the first stage ends with our study period (the most recent week). In general, all states in the first stage show higher variances than in the second stage. It implies that the public had been taking active responses, voluntarily staying home more, to the in-state confirmed cases even before mandatory orders were issued. After the stay-at-home mandates, both range and variance have become much tighter to the average value indicating that the mandates help closely stabilize the statewide movements. On the other hand, it should be acknowledged that other factors affecting these variations may exist, not only the stay-at-home orders. For instance, the public may be aware of higher infection risks with higher population density. In the next section, we further explore the mobility trends featured by state-level sociodemographic groups.

### Sociodemographic group trends

In this section, we demonstrate the mobility trends featured by sociodemographic groups. Median income level and population density show a notable clustering nature, which we choose to deliver in this paper. It is of great importance to mention that any features should not be singled out as the only contributing factor without theoretical grounds and rigorous research. Yet, several interesting findings are observed in relation to COVID-19.

**Fig 7:**
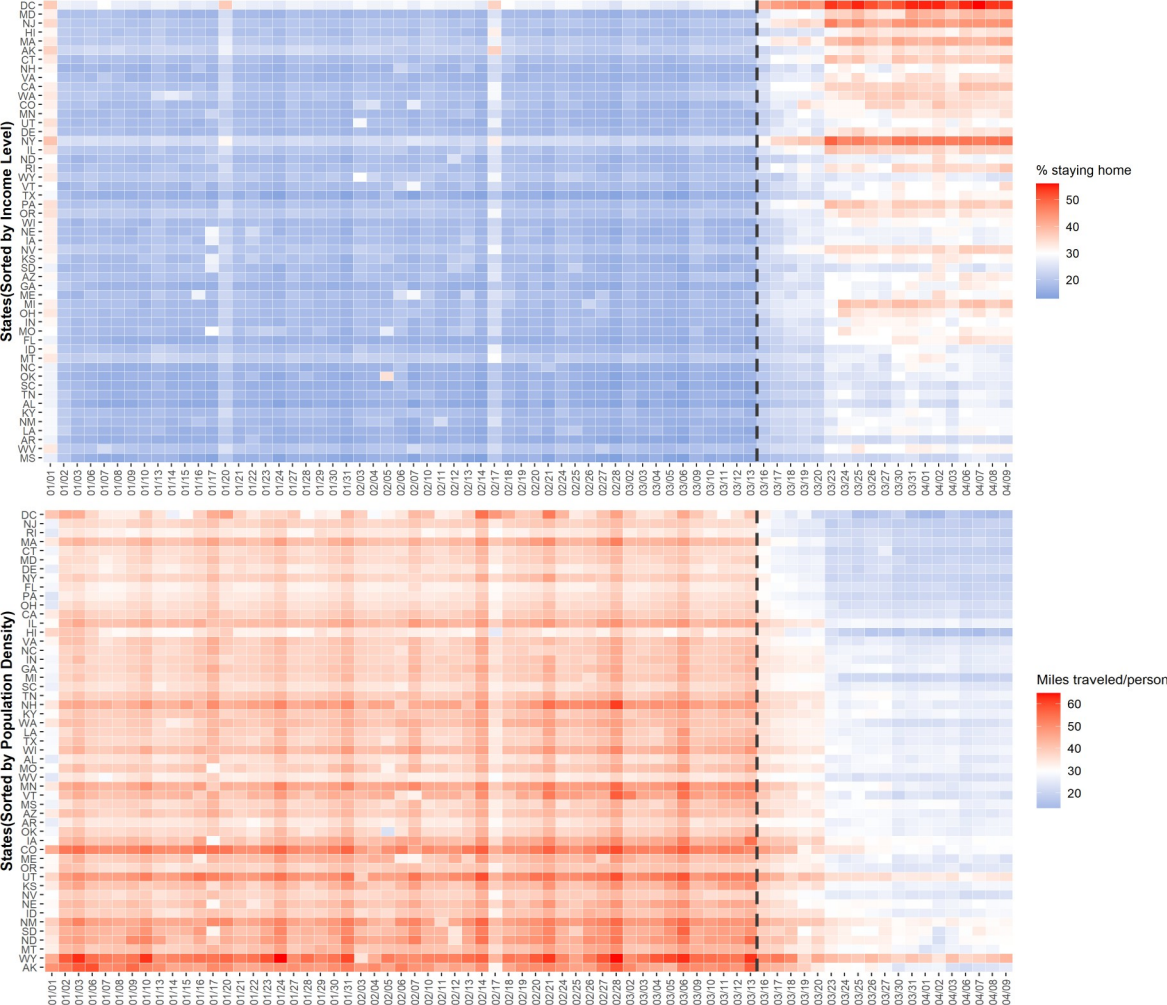
A statewide spatiotemporal comparison. (a) Percentage of people staying home by median income order (upper); (b) Miles traveled per person by population density order (lower).

Fig 7A illustrates the percentage of people staying home for income group comparison (upper), and Fig 7B demonstrates miles traveled per person in the order of population density (lower). The mobility pattern gradually becomes distinguishable between two groups upon the COVID-19 outbreak. Until the national emergency declared on March 13 (grey line), states share a relatively homogeneous staying home trend regardless of the sociodemographic features, while one can pinpoint a more distinctive trend after that. The higher income group tends to present a higher staying-at-home ratio and higher density group presents lower trip distance after the outbreak. Even though the income level or population density may not be the only factor that affects the mobility trend, there exists an overall heterogeneity between the groups.

**Fig 8:**
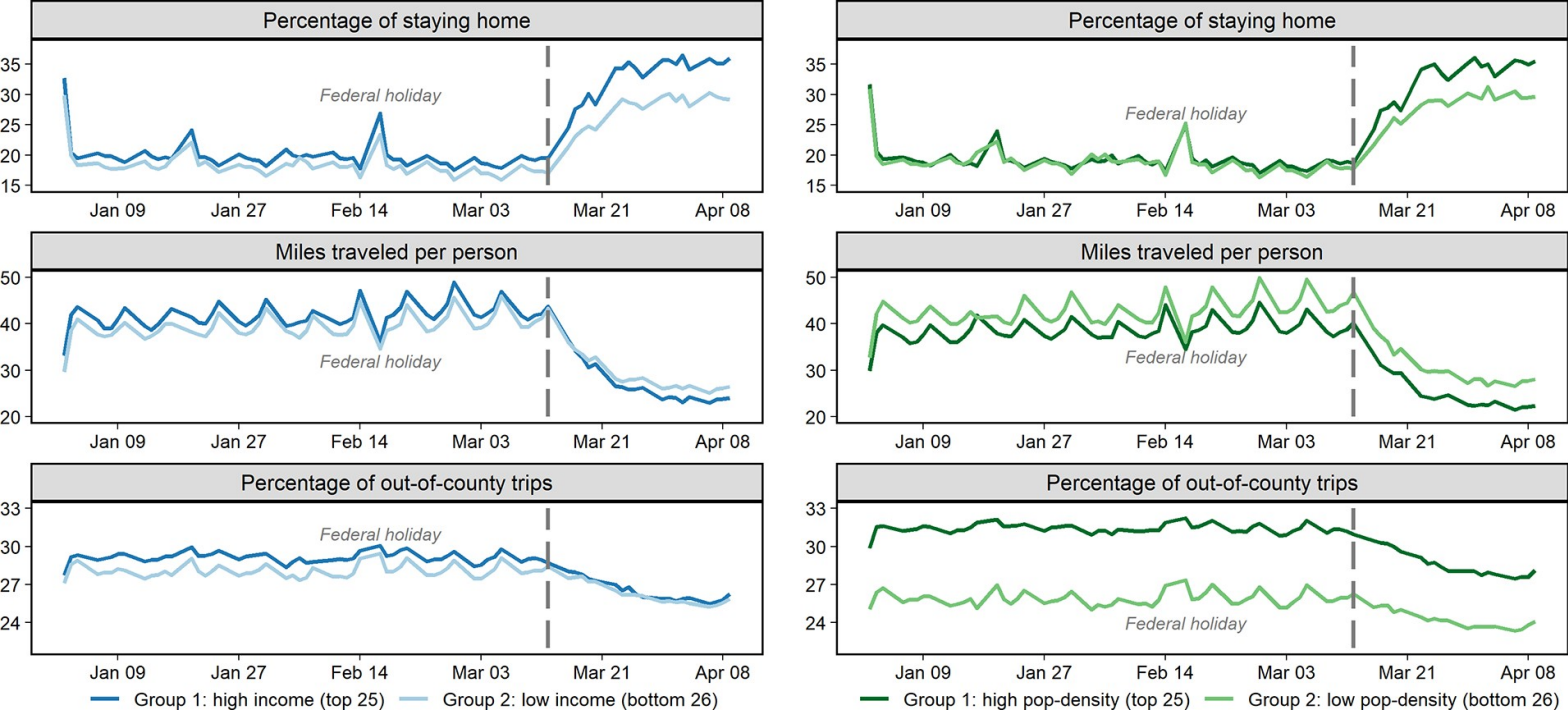
Groupwise mobility pattern comparison. (a) Median income groups (left); (b) Population density groups (right).

Fig 8 illustrates group mobility patterns in three measures. The upper box in Fig 8A indicates that the higher income states tend to stay at home more before the outbreak, but the gap becomes more notable afterward. It is noteworthy that, from the middlebox in Fig 8A, two income groups exchange their trip distance trend around mid-March (grey bar). The high-income group presents a tendency to travel slightly longer distances before the pandemic, while the low-income group surpasses later. The percentage of out-of-county trips relatively stays similar between the income groups, where the high-income group used to travel slightly more often to out-of-county destinations, compared to the population density groups in Fig 8B. However, it is still worth noting that the gap becomes diminished after mid-March. In terms of population density comparison in Fig 8B, the percentage of people staying home trend exhibits a similar pattern until mid-March (grey bar) between two groups. Afterward, the higher density group reduces their mobility more noticeably. One could conclude that the higher chances of contacting other people in the higher density area result in practicing social distancing more actively. At all times, the lower density states sustain higher miles traveled per person and lower out-of-country trip rates, which is agreeable considering lower density areas would have more scattered city shapes compared to an urban area. Unlike income comparison, no metrics exchange the trends over time but, instead, the gaps in miles traveled and out-of-county trips are more noticeable between groups throughout the study period.

### Teleworking trends

A teleworking rate is another crucial measure to reveal how many employees have been impacted by COVID-19. As discussed, the work trip rates have decreased amid the pandemic (-0.2 work trips per person) resulting from both teleworking and unemployment increases. In this section, we attempt to understand the increased rate of people staying home whether it is due to teleworking or unemployment by estimating the teleworking rate. This estimate is based on the work trip frequency per person and weekly unemployment claims from the United States Department of Labor [38]. For this demonstration, we first divide employees into two categories: teleworkers and commuters. Their initial relative ratio is estimated per state based on the American Community Survey (ACS) report [39]. The total number of employees (*teleworkers+commuters*) in the benchmark week are obtained from the weekly unemployment claims. The number of commuters in the benchmark week are estimated as the product of the commuter ratio and total employees. Assuming that the work trip rate per person is consistent within a short time period, we calculate the number of commuters by dividing the number of work trips by the work trip rate per person. Then, the number of teleworkers per week is estimated (*total employees−commuters−unemployed workers*) with an assumption that there is no additional weekly employment during COVID-19. Here, the number of weekly unemployed populations are estimated based on the unemployment claims.

**Fig 9:**
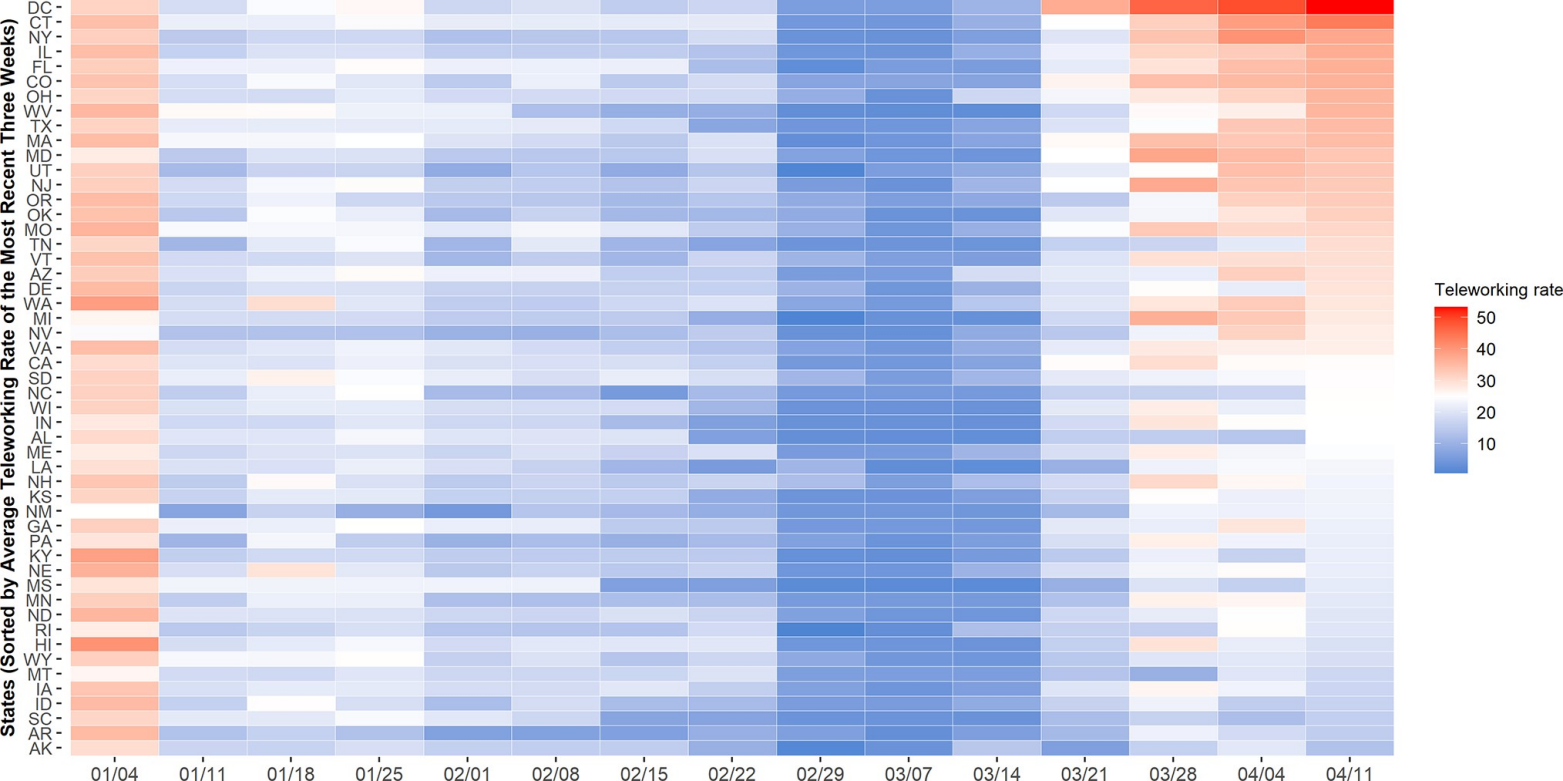
Weekly statewide teleworking trends for employees.

Despite our naive approach and assumptions, the estimates are in accordance with the pandemic circumstances. The teleworking rate begins to increase around mid-March complying with the sharp increase in COVID-19 cases. Fig 9 demonstrates the temporal variations where a baseline is set on the week of March 7 (Fig 9). As of April 4, the number of employees working from home increases by 25%, compared to the baseline week. The teleworking rate in some states stops increasing and even decreases in April. Two possible assumptions account for this trend: employees who no longer conduct teleworking and work on-site or employees who are no longer hired. One drawback to our estimation is that it does not separate the population who are on a break from teleworkers, which is the possible reason for the higher teleworking rate in the first week of January. Overall, we anticipate more research is invited to scrutinize this teleworking trend and rapidly inclining unemployment rates, which marks the highest increase in the seasonally adjusted series report history due to the pandemic [40]. Besides, as the teleworking trend has not been observed to this extreme extent before, further research will help provide essential evidence on the economic crisis.

## Findings

Understanding public reactions to the virus and the non-pharmaceutical interventions should be of great help to fight COVID-19 strategically. In order to provide an integrated perspective on public reactions related to the pandemic propagation and the non-pharmaceutical interventions, we examine day-by-day mobility variations across the U.S. by leveraging mobile device location data and the measures related to social distancing.

Based upon the noticeable degree of mobility trend alteration at a nation level, our study provides more detailed evidence on the mobility shifts from the statewide and sociodemographic group comparison. In addition, we attempt to estimate the teleworking population based on work trip rates integrated with unemployment claims data. Evidenced by our measures, a large number of populations have decreased their daily movements during the pandemic. This suggests that the government message urging to stay home is in line with public mobility behavior. Our findings can be summarized as follows, which are closely related to social distancing trends:

- the nationwide mobility has reduced amid the pandemic observed by all metrics;
- the declaration of the national emergency with the rapid increase in COVID-19 cases can be perceived as a significant stimulus to the increase in people staying home and decrease in mobility;
- the public has been taking active responses, voluntarily staying home more, to the in-state confirmed cases while the stay-at-home mandates stabilize the staying-at-home population with a smaller range of fluctuation;

- even though the income level or population density may not be the only factors that affect the mobility trend, there exists overall heterogeneity between the groups;
- income groups exchange the trend of miles-traveled-per-person measure, such that the higher income group tends to travel a longer distance before the pandemic while the low-income group surpasses the trend afterward;
- both before and after the outbreak, the lower density states sustain higher miles traveled and lower out-of-country trip rates and no metrics exchange the trends between two density groups;
- the gaps in miles traveled and out-of-county trips are more noticeable between the population density groups throughout the whole study period, compared to the income groups; and
- the teleworking trends are consistent with the pandemic circumstances, which begin to increase around mid-March inducing a 25% increase of teleworking employees by the first week of April compared to baseline.

One limitation of our study is that we examine a relatively early pandemic stage. Now with many more months into the pandemic from the study period, there could be an opportunity to find relationships between the continuous rising confirmed cases and the plateau of social distancing trends as well as the periodic trends of social distancing even after the state reopening. In addition, as we observe the clear evidence on social distancing efforts, a rigorous modeling approach will be necessary to quantify the social distancing practices and to analyze potential reasons behind. Also, our observation could be also integrated with pharmaceutical modeling research. While carefully suggesting the future research directions that can help the current and potential future matters, we anticipate our data-driven analysis offers integrated insights on social mobility trends regarding the pandemic circumstances. In light of bringing empirical evidence to bear on how people behave during the pandemic, this study could benefit to raise public awareness and, consequently, reinforce the importance of social distancing while assisting policymakers.

## Supporting information

S1 Appendix List of percentage changes in mobility in Fig 3 and Fig 4. (DOCX)
