# Supplementary material for: Human mobility trends during the early stage of the COVID-19 pandemic in the United States
Source: PLoS One. 2020 Nov 9;15(11):e0241468. doi: 10.1371/journal.pone.0241468 (PMC7652287; doi:10.1371/journal.pone.0241468)
Supplement: S1 Appendix — (DOCX) [file pone.0241468.s001.docx]

# S1 Appendix. List of percentage changes in mobility in Figure 3 and Figure 4.

| STNAME | Percentage of staying-at-home | | | Miles traveled per person | | |
| --- | --- | --- | --- | --- | --- | --- |
|  | Before vs. Benchmark | After vs. Benchmark | Recent vs. Benchmark | Before vs. Benchmark | After vs. Benchmark | Recent vs. Benchmark |
| AK | 41.2 | 51.5 | 47.9 | -37.1 | -48.8 | -52.7 |
| AL | 50.8 | 65.2 | 59 | -28.4 | -33.9 | -32.7 |
| AR | N/A | N/A | 43.3 | N/A | N/A | -27.2 |
| AZ | 54.8 | 67.5 | 67.5 | -29.9 | -36.4 | -36.9 |
| CA | 29.8 | 81.8 | 92.3 | -13.1 | -36.7 | -45.7 |
| CO | 50.8 | 68.9 | 62.5 | -30 | -43.1 | -43.5 |
| CT | 50.2 | 82.9 | 82.9 | -33.1 | -46.8 | -52.8 |
| DC | 82 | 84.4 | 83.9 | -57.3 | -60.2 | -59.4 |
| DE | 51.1 | 76.6 | 74.5 | -30.3 | -42.2 | -43 |
| FL | 85.1 | 100.4 | 96.9 | -37.8 | -44.2 | -43 |
| GA | 60.3 | 77.4 | 73 | -32.1 | -40.3 | -38.6 |
| HI | 43.9 | 78.2 | 74.7 | -26.5 | -52.2 | -55.2 |
| IA | N/A | N/A | 43.8 | N/A | N/A | -35.4 |
| ID | 23.4 | 49.7 | 40.7 | -16.3 | -33 | -32.1 |
| IL | 30.5 | 81.2 | 70.8 | -17.8 | -40.4 | -42 |
| IN | 38.5 | 63.6 | 54.5 | -21.4 | -37.9 | -37.2 |
| KS | 53.2 | 61.4 | 59.3 | -35.4 | -40.4 | -39.5 |
| KY | 41.7 | 47.2 | 45.8 | -25.8 | -33 | -34.8 |
| LA | 39.2 | 69.2 | 70.8 | -19.1 | -32.2 | -36.1 |
| MA | 57.1 | 84.2 | 83.6 | -36.7 | -51 | -53.4 |
| MD | 91.4 | 105.8 | 102.9 | -45.3 | -50.5 | -50 |
| ME | 41 | 44.3 | 44.3 | -35.1 | -40.9 | -40.9 |
| MI | 44.3 | 91.8 | 86.3 | -27.2 | -49.4 | -50.4 |
| MN | 57.2 | 79.6 | 69.1 | -35.6 | -48.1 | -46.2 |
| MO | 53.4 | 46.6 | 56.1 | -33.9 | -29 | -35.3 |
| MS | 45.1 | 64.4 | 59.5 | -25.6 | -34.2 | -32 |
| MT | 20.5 | 37.6 | 28.4 | -24 | -36.1 | -33.8 |
| NC | 49.8 | 60.5 | 55.9 | -31.7 | -37.1 | -36.4 |
| ND | N/A | N/A | 54.5 | N/A | N/A | -39.4 |
| NE | N/A | N/A | 53.2 | N/A | N/A | -35.5 |
| NH | 48.9 | 65.2 | 59.6 | -35.5 | -45.1 | -45.6 |
| NJ | 49.2 | 107.2 | 107.2 | -26.8 | -51.1 | -53 |
| NM | 27.3 | 54.7 | 51.1 | -16.9 | -33.1 | -35 |
| NV | 67.2 | 73.1 | 73.1 | -35.1 | -41.1 | -41.4 |
| NY | 42.4 | 85.3 | 87 | -25.7 | -48.1 | -52.5 |
| OH | 38.4 | 68.5 | 61.6 | -23.8 | -40.9 | -40.1 |
| OK | 34.5 | 44.7 | 54.2 | -14.4 | -26.2 | -30.7 |
| OR | 28.4 | 64.5 | 55.5 | -17.5 | -38 | -40.3 |
| PA | 79.4 | 75.4 | 75.4 | -43.2 | -44 | -43.9 |
| RI | 60.4 | 79.5 | 78.2 | -37.8 | -46.1 | -47.8 |
| SC | 47.8 | 46.6 | 49.4 | -31.6 | -29.2 | -32.8 |
| SD | N/A | N/A | 39.9 | N/A | N/A | -33.5 |
| TN | 48.5 | 55.3 | 56.1 | -28.4 | -33.2 | -33.9 |
| TX | 82.1 | 90.2 | 90.2 | -34.1 | -37.7 | -37.7 |
| UT | N/A | N/A | 52.7 | N/A | N/A | -34.4 |
| VA | 71.4 | 81.7 | 79.5 | -38.4 | -43.2 | -43.2 |
| VT | 31.6 | 50 | 47.4 | -30.4 | -45.5 | -45.4 |
| WA | 42.9 | 82.3 | 72.8 | -24.9 | -44.5 | -46.9 |
| WI | 47.6 | 63.6 | 54.5 | -26.4 | -40.5 | -39.2 |
| WV | 25.9 | 43.8 | 41.9 | -20.6 | -32.4 | -33.6 |
| WY | N/A | N/A | 22.7 | N/A | N/A | -21.1 |

Note: ‘N/A’ refers to no stay-at-home order implemented; ‘Before’ refers to a week before the order date and ‘After’ refers to a week after the order date, which vary for each state; ‘Benchmark’ period is from February 3 to February 16; ‘Recent’ period is from April 6 to April 12
